# Comprehensive Analyses of Serine Protease-like Protease (SBT) in Regulating Yield Characters in Rapeseed (*Brassica napus* L.)

**DOI:** 10.3390/plants15091318

**Published:** 2026-04-25

**Authors:** Xiangtian Shi, Qian Lei, Sirou Xiang, Kun Lu, Cunmin Qu, Jiana Li, Liyuan Zhang

**Affiliations:** 1 Integrative Science Center of Germplasm Creation in Western China (CHONGQING) Science City, College of Agronomy and Biotechnology, Southwest University, Beibei, Chongqing 400715, China; niplan521@email.swu.edu.cn (X.S.); layxiiiiiiii5757@email.swu.edu.cn (Q.L.); drqucunmin@swu.edu.cn (C.Q.); ljn1950@swu.edu.cn (J.L.); 2 Chongqing Three Gorges Academy of Agricultural Sciences, Wanzhou, Chongqing 404155, China; xsr961103@163.com

**Keywords:** *Brassica napus* L., SBT, phylogenetic analysis, expression pattern, yield characters

## Abstract

Serine protease-like proteases (SBTs) constitute a distinct class of serine proteases exclusive to plants. Despite the recognized importance of SBTs in various plants, knowledge concerning the evolution and function of *SBT* genes in *Brassica napus* is limited. In this study, a total of 140, 63, and 71 *SBT* genes were identified in *B. napus*, *B. oleracea*, and *B. rapa*, respectively. Phylogenetic analysis classified these 330 identified SBTs into five subfamilies, and collinearity analyses further indicated that gene redundancy and gene loss were strongly associated with polyploidization in Brassicaceae plants. Additionally, analyses of gene structure and conserved motifs suggested that evolutionary changes in exon-intron structures may contribute to the differentiation of coding regions, expression patterns, and even functions within the *BnSBT* family. Analysis of promoter *cis*-regulatory elements revealed their predominant association with hormonal responses, abiotic stress, and processes related to plant growth and development. Furthermore, eight differentially expressed genes (DEGs) were identified through a comparative analysis of RNA-Seq data from high- and low-yielding cultivars. qRT-PCR verification also revealed that these eight DEGs (*BnSBT1.4b*, *BnSBT1.4c*, *BnSBT1.4d*, *BnSBT1.5c*, *BnSBT1.6b*, *BnSBT1.8a*, *BnSBT3.14a*, and *BnSBT3.14b*) were significantly differentially expressed in the pericarp and seeds. They could be categorized into two distinct groups: *BnSBT1.4b*, *BnSBT1.4c*, *BnSBT1.4d*, *BnSBT1.5c*, and *BnSBT1.8a* were highly expressed in high-SPSI material, whereas *BnSBT1.6b*, *BnSBT3.14a*, and *BnSBT3.14b* were highly expressed in low-SPSI material. These results suggest that *BnSBTs* have diverse potential functions in regulating yield traits in *Brassica napus*. These findings offer key insights into Brassicaceae *SBT* genes and highlight the importance of *BnSBTs* in achieving high yields in *Brassica napus*.

## 1. Introduction

The normal function of plant cells depends on the meticulous equilibrium between protein synthesis and degradation [1], which requires the participation of various proteases. Most plant proteases fall under the category of serine proteases, and the subtilisin-like protease (SBT) family from *Bacillus cereus* is the second largest within the serine protease family. Hence, investigating the *SBT* gene family is vital for gaining a comprehensive insight into key areas of plant biology, such as growth, development, and environmental adaptation [2,3].

The origin of *SBT* can be traced back to the melon (*Cucumis melo*), from which the first *SBT* was isolated and identified [4]. *SBT* gene families with considerable diversity have since been discovered in a range of plant species, including tomatoes [5], grapes [6], rice [7,8], and cotton [9,10]. The presence of *SBT* families suggests a broad distribution and diversity in plants. Through extensive evolutionary analysis, it has been revealed that plant subtilisins underwent numerous gene duplication events throughout their prolonged evolutionary history, resulting in an increased spectrum of functionalities [11]. By systematically analyzing the evolution of the entire *SBT* gene family across archaea, bacteria, and eukaryotes, it was revealed that plant subtilisin originated from bacteria through a horizontal gene transfer (HGT) event, infiltrated into fungal and algal lineages, and subsequently experienced gene duplication events in the common ancestor of leguminous algae and embryophytes [12]. From a structural standpoint, SBTs demonstrate remarkable conservation, showcasing the peptidase_S8 domain with a distinctive arrangement of catalytic triad amino acid residues—Asp, His, and Ser [13,14]. Moreover, several SBTs are reported to harbor proteinase-associated (PA) domains and C-terminal protein (Fn) III-like domains, which play pivotal roles in modulating selective substrates and protease activity [15].

Currently, the *SBT* gene family has been studied across diverse plant species [3]. In *Arabidopsis*, a comprehensive tally of 56 *SBT* members has been identified [16]. Members of SBT1 play irreplaceable roles, among which the *AtSBT1.1* gene can promote cell division in the root meristem and fine-tune immune responses [17]. *ATSBT1.2* chiefly regulates stomatal density [16]. *ATSBT1.4* is involved in reproductive development and impacts seed yield by affecting silique abundance [18]. Additionally, *ATSBT1.4* is intricately linked to ABA signaling in increasing plant resistance to drought [19]. *AtSBT1.7* profoundly affects seed mucilage de-esterification and the inhibition of germination under conditions of limited water availability [20]. Moreover, *SBT* genes have been reported in other species. In soybeans, *GmSBT* exhibits distinctive substrate specificity, catalyzing the breakdown of storage proteins within soybean seeds. In cotton, *GbSBT1* intricately affects plant growth and fruit yield [9]. In pineapples, *AcoSBT1.12* overexpression results in a postponement of flowering [21]. In total, SBTs constitute a proteinase family that exerts extensive influence in plant biology, where their multifaceted functionalities and structural conservatism underscore their pivotal roles in governing various cellular activities and physiological processes. However, no publication has reported *SBT* genes in *Brassica napus*.

Rapeseed (*B. napus*; AACC, 2*n* = 38) represents a polyploid species resulting from the hybridization of turnip rape (*B. rapa*, AA, 2*n* = 20) and wild cabbage (*B. oleracea*, CC, 2*n* = 18), merging the A and C genomes [22,23,24,25]. Previous research has demonstrated that the *Brassica napus* genome experienced substantial duplication events over the course of its evolution [26,27], posing significant challenges for the functional study of individual genes [28]. Therefore, evolutionary analysis of candidate yield genes is of great significance for future high-yield molecular breeding, as homologous copies can be manipulated through gene editing. *SBTs* have been reported to be yield-determining genes in many species [29,30,31] (the majority of *SBT* genes are involved in multiple stages of plant signal transduction, including embryonic development, seed maturation, lateral root formation, root tip division, stomatal opening, and reproductive development). However, no publication has reported on the main Brassicaceae species.

Thus, this study performed a genome-wide analysis to identify *SBT* genes across the four principal species within the Brassicaceae family. Following phylogenetic analysis, the 330 identified *SBT* genes were classified into five subgroups. Based on analyses of the physicochemical properties and expression patterns of the 140 identified *BnSBTs*, significant diversity was observed among subgroup members. Meanwhile, different homologous copies within the same subfamily were significantly conserved. Additionally, to clarify whether *BnSBTs* are related to yield characteristics in *B. napus*, we conducted RNA-Seq and qRT-PCR analyses on the expression patterns of the 140 *BnSBTs* among 12 different yielding cultivars, and eight differentially expressed genes (DEGs) were ultimately identified. In summary, this study comprehensively analyzed the *SBT* genes and provided a theoretical basis for future functional research on key candidate *BnSBTs*.

## 2. Results

### 2.1. Identification and Phylogenetic Analysis of SBTs

**Figure 1:**
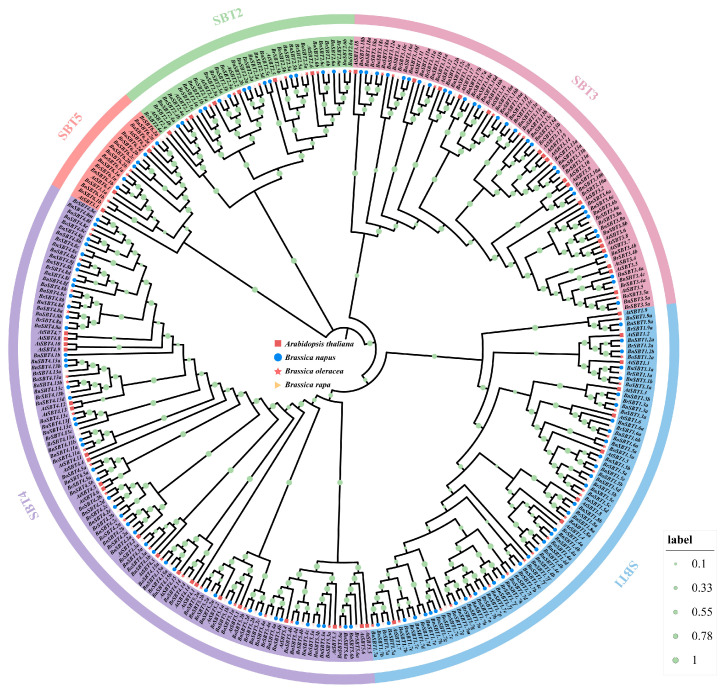
Neighbor-joining (NJ) tree of SBT protein sequences from *Brassica napus*, *Brassica rapa*, *Brassica oleracea*, and *Arabidopsis thaliana*. Different colors and labels represent specific subfamilies of SBT proteins, including SBT1, SBT2, SBT3, SBT4, and SBT5. The bootstrap values are indicated as green dots along the branches, with the size corresponding to the confidence level.

To better understand the *SBT* genes, we performed evolutionary analysis. Based on the 56 *AtSBT* protein sequences [16], 140 *BnSBT*, 71 *BrSBT*, and 63 *BoSBT* genes were identified through BLASTP (v2.14.0) and HMM (v3.0) analyses (Table S1). Phylogenetic analysis was conducted on 330 total protein sequences from the four main Brassicaceae species, and the results revealed that the 330 *SBT* genes were classified into five subfamilies: SBT1, SBT2, SBT3, SBT4, and SBT5 (Figure 1). Moreover, the copy numbers of *SBT*s between *Arabidopsis* and *Brassica napus* varied from 1 to 16. However, it is worth noting that only 43 *AtSBT*s matched with *BnSBT* genes, whereas the remaining 13 *AtSBT*s lacked homologs in *Brassica napus*, *Brassica oleracea*, and *Brassica rapa* (Table S2). These results indicate that some genes have been lost or duplicated during genome evolution.

### 2.2. Chromosome Locations of BnSBTs and Duplication Analysis

**Figure 2:**
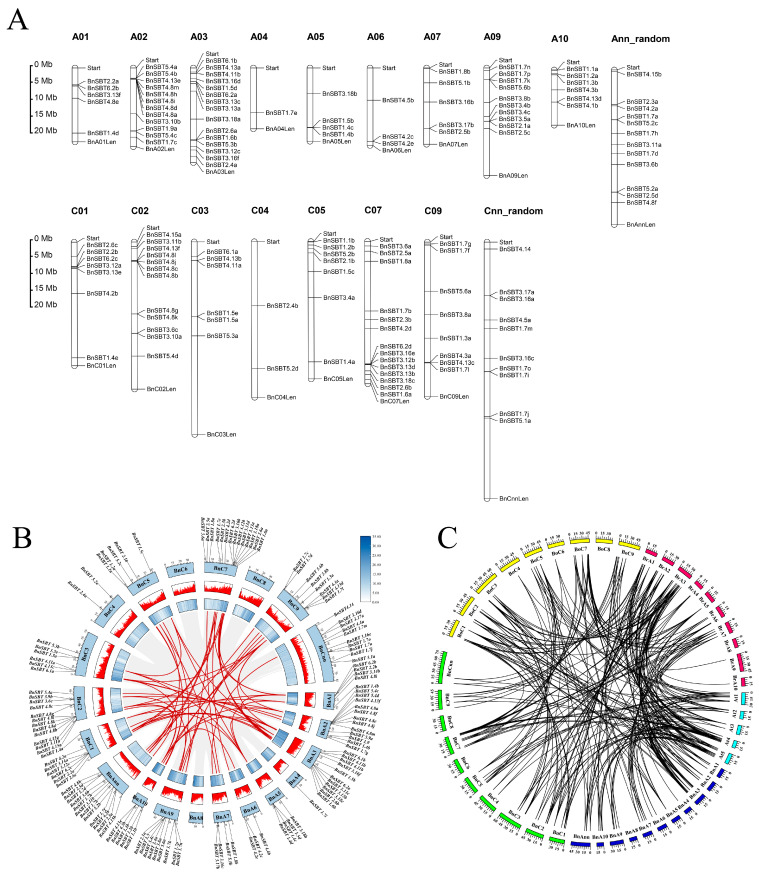
Chromosome locations of the 140 *BnSBTs* and syntenic analysis among the 330 *SBT* genes. (**A**) Distribution of the 140 *BnSBT*s on the chromosomes. The chromosomes are numbered A01–A10 and C01–C09, and Ann random and Cnn random represent randomly distributed pseudochromosomes. Chromosome length is designated by the labels “Start” and “Len”, with units in megabases (Mb). (**B**) Chromosomal distribution and duplication events of *SBT*s in *B. napus*. Chromosome numbers and *BnSBT* gene locations are depicted in the outer circle, and the inner bar plots indicate gene density or specific gene expression levels. Red lines within the circle denote tandem or segmental duplication events. The specific values correspond to the blue gradient as shown in the inset. (**C**) Syntenic relationships of *SBT* genes among the four main Brassicaceae crops. The outer circle represents different chromosomes; dark blue and green indicate the A and C subgenomes of *B. napus*, respectively; red and yellow represent *B. rapa* and *B. oleracea*, respectively; and sky blue represents *A. thaliana*. Black lines indicate syntenic gene pairs.

Among the 140 *BnSBT*s, 118 are unevenly distributed on 19 chromosomes, including 61 genes assigned to the A subgenome and 57 to the C subgenome, whereas the remaining 22 *BnSBT*s are currently unknown (Figure 2A). In detail, chromosomes A02, A03, A09, C02, C07, and C09 each contain more than 10 *BnSBT*s; in contrast, chromosomes A04, A05, A06, A07, and C04 each have fewer than 6 *BnSBT*s. Moreover, some genes, such as *BnSBT5.4a* and *BnSBT5.4b* (A02), *BnSBT3.13a* and *BnSBT3.13c* (A03), *BnSBT1.4b* and *BnSBT1.4c* (A05), *BnSBT4.2c* and *BnSBT4.2e* (A06), *BnSBT1.5a* and *BnSBT1.5d* (C03), *BnSBT3.13b* and *BnSBT3.13d* (C07), and *BnSBT1.7g* and *BnSBT1.7f* (C09), are closely linked on the same chromosome, suggesting the occurrence of tandem duplication within the *SBT* gene family throughout genome evolution. In Figure 2B, lines denote the syntenic relationships among the 140 *BnSBT* genes, from which 78 homologous gene pairs were identified (Figure 2B and Table S4).

Moreover, colinear regions containing *SBT* genes were found among the main Cruciferous species (Figure 2C and Table S5). In *Arabidopsis*, *SBT* colinear genes are located primarily on chromosomes A4 and A5; in *Brassica rapa*, they are found mainly on chromosome A03; and in *Brassica oleracea*, they are concentrated on chromosomes C07 and C09. In contrast, *SBT* colinear genes in *Brassica napus* primarily reside on chromosomes A03 and C07. However, there are differences in the distribution of *SBT* colinear genes between *Brassica napus* and its parent species, suggesting that the *Brassica napus* genome underwent a certain degree of reorganization following hybridization.

### 2.3. Exon–Intron Structures and Conserved Motif Analysis of the BnSBTs

**Figure 3:**
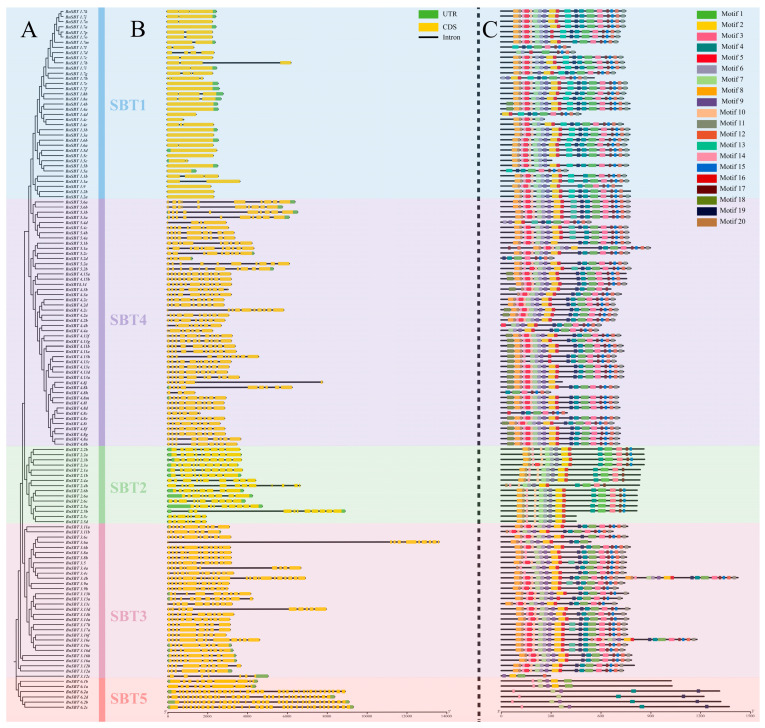
Analysis of the phylogenetic relationships, conserved motifs, and gene structures of the 140 identified *BnSBT*s. (**A**) Phylogenetic tree of SBT gene family members. Different subfamilies are shaded with distinct background colors. All 140 *BnSBTs* were confirmed as non-pseudogenes with intact open reading frames (ORFs) during annotation (Table S1). (**B**) Gene structures of the 140 *BnSBT*s. Gene lengths are scaled proportionally along the horizontal axis. (**C**) Conserved motifs of BnSBT proteins. Motif numbers (1–20) are indicated.

To gain deeper insights into the protein profiles, exon–intron structures, and conserved motifs of the 140 *BnSBTs* were further examined (Figure 3). Analysis showed that exon numbers varied from 1 to 34, while intron counts ranged from 0 to 33. Significant differences in gene structure were observed among subgroups, although genes within the same subgroup generally shared comparable structural architectures, with a few exceptions (Figure 3B). Among all analyzed genes, members of the SBT1 subfamily had fewer exons and introns, whereas genes in other subfamilies typically had eight or more exons. Similarly, the motif distribution among different phylogenetic subgroups was similar to that of the gene structures (Figure 3C). However, some genes, such as *Bna.SBT1.4b* and *Bna.SBT1.4c* exhibited fewer motifs due to deletions at the C- or N-terminus. In the SBT3 subgroup, the motif arrangement of different genes was highly similar. While widely distributed, Motifs 1 and 2 were absent in certain shorter genes, including *Bna.SBT1.4b*. Furthermore, the BnSBT proteins exhibited a broad range of amino acid lengths, spanning from 217 to 1424 residues, with molecular weights ranging from 11.4 to 152.2 kDa and isoelectric points ranging from 5.03 to 9.58 (Table S3). Based on subcellular localization predictions, the majority of proteins were predicted to reside in the cell wall. Exceptions included *BnSBT1.7i* (cell membrane), *BnSBT2.4a* (nucleus), and *BnSBT6.2d* (chloroplast) (Table S3).

In summary, the consistency of gene organization within the same subgroup was supported by phylogenetic, motif, and structural analyses. This stability in amino acid conservation among SBT proteins implies that closely related members within a phylogenetic clade are likely to perform analogous functions.

### 2.4. Analysis of Cis-Regulatory Elements with the Promoters of 140 BnSBTs

**Figure 4:**
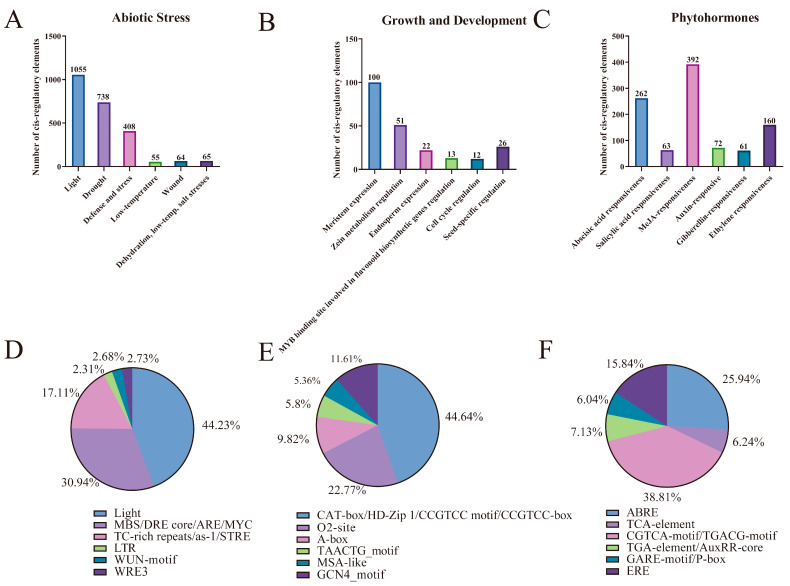
Analysis of promoter *cis*-elements in *BnSBTs*. (**A**–**F**) Bar charts and pie charts, respectively, illustrate the total counts and percentage distributions of *cis*-elements associated with abiotic stress (**A**,**D**), growth and development (**B**,**E**), and phytohormones (**C**,**F**). Different colors represent distinct *cis*-elements and their proportions across the *BnSBTs*.

In an effort to elucidate the functions of *BnSBTs* in plant growth, development, and phytohormone responses in *B. napus*, our analysis focused on three principal classes of cis-regulatory elements: those involved in abiotic stress responsiveness, phytohormone responsiveness, and growth and development regulation (Figure 4 and Figure S1). In terms of abiotic stress responsiveness, the main elements included light-responsive, drought-responsive, and low-temperature-responsive elements (Figure 4D). Regarding phytohormone responsiveness, a total of 1010 elements were identified. Among them, abscisic acid (ABA)-related accounted for 25.94%, and MeJA-related accounted for 38.81%. Within the *BnSBT* promoter regions, 107 gene promoters contained ABA-related elements, and 96 contained MeJA-related elements (Table S6). Additionally, elements related to salicylic acid, auxin, gibberellin, and ethylene were detected (Figure 4C,F). Analysis identified six types of *cis*-elements associated with growth and development regulation, with CAT-box (44.64%) and O2-site (22.77%) being the most prevalent, which are associated with meristem expression, cell cycle control, and seed-specific regulation (Figure 4E).

Overall, these results suggest that *cis*-regulatory elements in *BnSBT* genes are widely distributed across multiple genes, with some elements being gene-specific. Their expression patterns may vary depending on developmental stage, phytohormone signaling, or abiotic stress conditions.

### 2.5. Preliminary Identification of Major Candidate Key Yielding BnSBTs

Investigation of the expression profiles of the 140 *BnSBT* genes, based on RNA-Seq data from 36 tissues of the ZS11 material obtained from an online database (brassica.biodb.org/index, accessed on 20 August 2023), showed a strong correlation with their subfamily classification. Most notably, genes from the SBT1 subgroup were highly expressed in multiple tissues, especially *BnSBT1.4*, *BnSBT1.5*, *BnSBT1.6*, *BnSBT1.8, and BnSBT1.7a/b* (Figure S2). Several genes from the SBT2 and SBT5 subfamilies were expressed only in specific tissues. In contrast, *SBT4s* were expressed at low levels in all tissues. Moreover, different expression levels also occurred within the same subfamily. For example, *BnSBT1.7e* and *BnSBT1.7f* were highly expressed in all tested tissues, and the genes *BnSBT1.7i/j/k/m/n* were expressed at relatively high levels in pericarps. Meanwhile, the other *BnSBT1.7* genes were expressed at very low levels. Overall, the SBT1, SBT2, and SBT5 subfamily genes exhibited broad expression patterns across multiple tissues, which may be related to their diverse biological functions.

**Figure 5:**
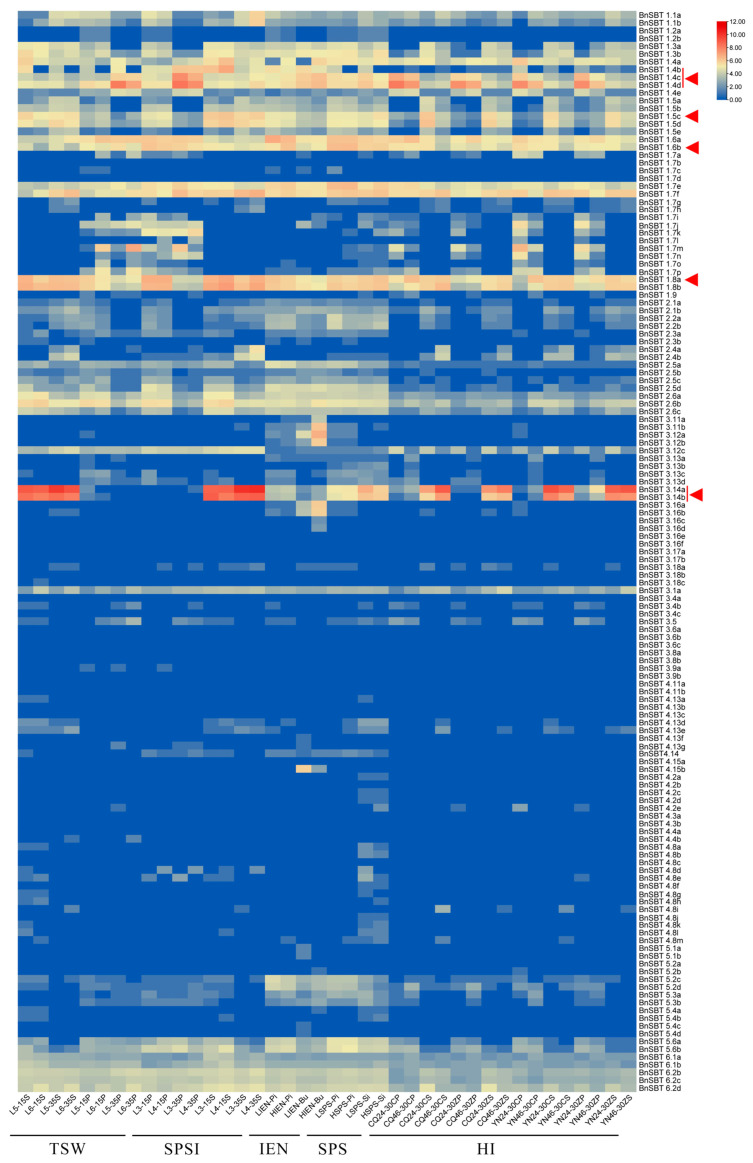
Expression patterns of the 140 *BnSBT* genes between yield-related extreme materials. L5: low thousand-seed weight (TSW); L6: high thousand-seed weight (TSW). L3 and L4: low and high seed weight per silique index (SPSI); LIEN: low initial embryonic number (IEN); HIEN: high initial embryonic number (IEN). LSPS and HSPS: low and high seed number per silique; CQ and YN indicate different cultivation locations. CQ24 and YN24 are materials with high harvest index (HI) values, whereas CQ46 and YN46 are materials with low harvest index (HI) values. Abbreviations for tissues: J: Stem; S: Seed; P: Silique pericarp; C: Lateral branch; Z: Main inflorescence; L: Flower bud; Pi: Pistil; Si: Silique. Red triangles represent the positions of the eight key candidate differentially expressed genes.

To screen key candidates yielding *BnSBTs*, we further analyzed the RNA-Seq data between materials with extremely high and low yields (Figure 5). Coincidentally, the overall expression patterns of the 140 *BnSBTs* exhibited high degrees of similarity to those in the ZS11 material. As shown in Figure 4, compared with the other subfamily members, only the *BnSBT1* gene was expressed at higher levels in four pairs of yielding cultivars, including materials with extreme phenotypes for TSW, IEN, SPSI, and SPS (all the phenotypic characteristics are described in detail in our previous publication [32]). Considering the significant differential expression of *BnSBTs* across multiple extreme materials with yield-related traits, we analyzed the expression profiles of 140 *BnSBTs* using harvest index (HI, a quantitative yield-related trait) (RNA-Seq data published in our previous study [33]). Ultimately, eight genes (*BnSBT1.4b*, *BnSBT1.4c*, *BnSBT1.4d*, *BnSBT1.5c*, *BnSBT1.6b*, *BnSBT1.8a*, *BnSBT3.14a*, and *BnSBT3.14b*) that showed consistent differential expression in both high- and low-yielding extreme materials were ultimately selected as key candidate differentially expressed genes (DEGs) for further verification (indicated with red triangles in Figure 5).

### 2.6. qRT-PCR Verification of the Identified Differentially Expressed Genes

**Figure 6:**
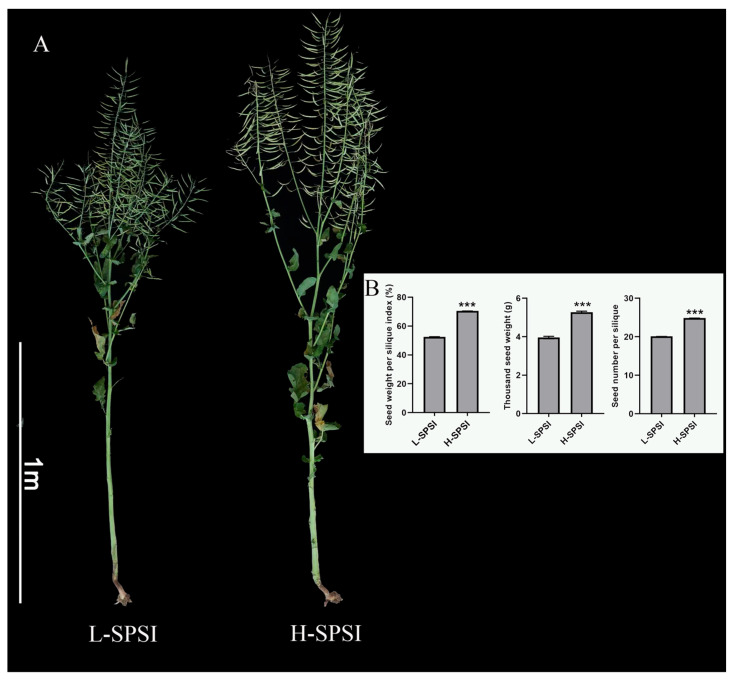
Phenotypic analysis of the L-SPSI and H-SPSI materials of *Brassica napus*. (**A**) L-SPSI represents low-yield material with a seed weight per silique index (SPSI), and H-SPSI indicates high-SPSI material. Bar = 1 m. (**B**) Quantitative comparison of silique-related yield traits between L-SPSI and H-SPSI. Data are presented as the mean ± SE. *** indicates a statistically significant difference (*p* < 0.001) between L-SPSI and H-SPSI as determined by Student’s *t*-test.

**Figure 7:**
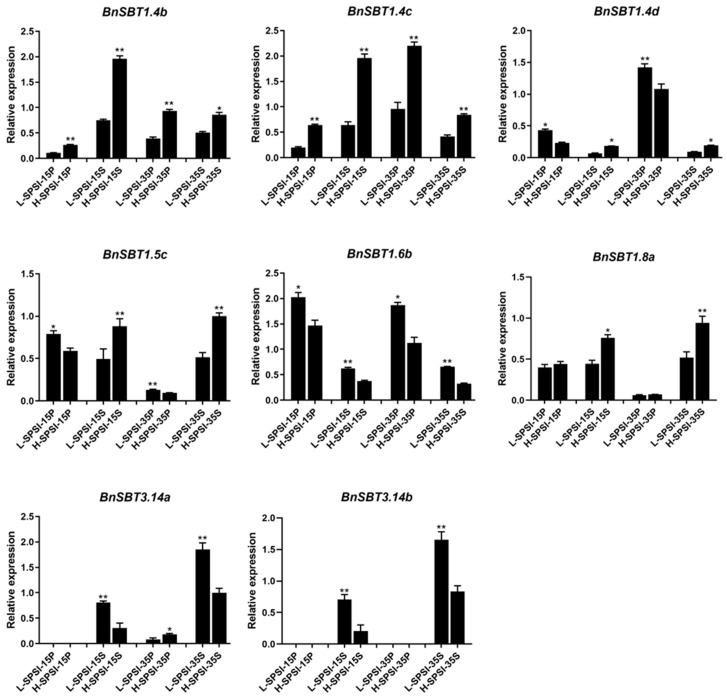
qRT-PCR verification of the identified differentially expressed genes (DEGs) between materials L-SPSI and H-SPSI. L-SPSI and H-SPSI represent low- and high-yield-related materials, respectively. 15/35P and 15/35S represent silique pericarp and seed samples collected at 15 and 35 days after flowering, respectively. *: significant difference with *p* value < 0.05; **: significant difference with *p* value < 0.01.

To verify whether the DEGs selected from the RNA-Seq analysis were significantly differentially expressed in yield materials, we performed qRT-PCR between two lines of cultivars with extremely high and low SPSI. This comprehensive trait effectively reflects rapeseed yield. Further quantitative comparisons of key yield-related traits revealed that H-SPSI resulted in significantly greater values than L-SPSI did in terms of SPSI, TSW, and SPS (*p* < 0.001) (Figure 6B and Table 1). All the plant morphology and phenotypic data are presented in Figure 6. The qRT-PCR results revealed that these DEGs exhibited tissue- and stage-specific expression (Figure 7 and Table S8). The eight DEGs could be categorized into two groups: *BnSBT1.4b*, *BnSBT1.4c*, *BnSBT1.4d*, *BnSBT1.5c*, and *BnSBT1.8a* were highly expressed in high-SPSI material, whereas the genes *BnSBT1.6b*, *BnSBT3.14a*, and *BnSBT3.14b* were highly expressed in low-SPSI material (Figure 7). Given that the development of both the pericarp and the seed is crucial for seed formation, the differential expression patterns of these genes suggest potential roles in silique structure formation and developmental regulation.

## 3. Discussion

### 3.1. Diversity and Conservation of the 140 Identified BnSBTs

Rapeseed (*B. napus*, AACC, 2*n* = 38) is a typical allotetraploid crop derived from the fusion of *Brassica rapa* (*n* = 10) and *Brassica oleracea* (*n* = 9) [34]. With the release of whole-genome sequencing data for rapeseed in 2014 [35], the origin and evolution of rapeseed have become a popular research topic in the past decade. Recently, a large number of gene families have been revealed through whole-genome analysis for their evolutionary status in *B. napus*, such as MST [26], SWEET [36], and TPS [37]. Similarly, some *SBTs* have been identified in many plants [10,31,38,39]; however, there are no reports of *SBT* identification in *B. napus*. In this study, 56, 63, 71, and 140 *SBT* genes were identified in *A. thaliana*, *B. oleracea*, *B rapa*, and *B. napus*, respectively (Table S1). Based on previous research, each *Arabidopsis* gene has approximately 4.4 homologs in the *Brassica napus* genome [40]. However, we found that each *AtSBT* has 1–16 homologs in *Brassica napus* (Table S2), which may be attributed to genome contraction and redundancy [41,42]. Additionally, the total number of *BoSBTs* (63) and *BrSBTs* (71) is lower than the 140 *BnSBTs* identified, indirectly indicating potential redundancy among *BnSBT* genes. Previous studies have shown that gene redundancy and loss can promote survival and adaptation in rapidly changing environments by increasing genomic plasticity and genotypic diversity [43,44]; therefore, the variation in gene copy numbers highlights the complex process of gene retention and loss, reflecting the complexity and diversity of genome evolution in *B. napus* [25,45].

Gene function may be largely determined by its structure [46,47]. Significant structural differences were observed among the 140 *BnSBTs*, with greater variation among subfamilies, as revealed by comprehensive analyses of gene structure and physicochemical profiles (Figure 3 and Table S3). For instance, compared with the other subgroup genes, all *SBT1* genes contain fewer exons and introns, such as *BnSBT1.2a* and *BnSBT1.2b*, which each contain only one exon. In contrast, most members of the other four subfamilies each possess more than 8 exons and introns (Figure 3). Previous studies have shown that variations in exon–intron structures can alter coding regions, thereby affecting gene expression and function [48,49,50]. Coincidentally, we also found that only *BnSBT1s* had higher expression levels in all 36 tested tissues in the ZS11 cultivar (Figure S2). All these results indicate significant diversity among the five subfamilies of the *BnSBTs*, including gene structure and expression patterns. Conversely, gene structures are relatively similar within the same subfamily (Figure 3). Similarly, most *BnSBT* genes within the same subfamily exhibited similar expression patterns (Figure 5 and Figure S2). These findings indicate the high conservation of gene structure and expression profiles within the same subgroups. In summary, differences in gene structure may cause changes in protein structure, affecting gene expression levels and resulting in functional differences, which help plants adapt to the environment and perform diverse functions during development [51,52].

### 3.2. High Possibility of Partial BnSBTs in Regulating Yield Traits in B. napus

Serine protease-like protease (SBT) genes have been shown to play a pivotal role in the regulatory network governing yield traits, such as the seed setting rate, seed number, and seed weight, by modulating storage protein utilization, plant structural development, and seed formation. Therefore, they are closely associated with the final plant yield [53,54,55]. In this study, we performed a comprehensive analysis to determine whether *BnSBTs* are associated with rapeseed yield. First, the analysis of the *cis*-regulatory elements in their promoters revealed that these elements are associated with plant hormones and plant growth and development (Figure 4 and Figure S1). Hormone-responsive elements such as ABA, JA, and IAA are widely present in *BnSBT* genes, suggesting their involvement in growth regulation [56,57,58,59]. Moreover, subcellular localization analysis revealed that 137 BnSBT proteins are predicted to be localized in the cell wall (Table S3), which plays important roles in regulating cell elongation and division and other processes [60]. Additionally, cell wall proteins can act as signaling molecules or receptors involved in signal transduction and in perceiving and responding to pathogen invasion, thereby activating plant defense mechanisms [61,62]. Studies have shown that these proteins also participate in the transport of nutrients and information molecules through the apoplastic pathway [63,64].

Both the RNA-Seq data and qRT-PCR verification revealed that some *BnSBTs* exhibited significant differential expression specificity among different yielding cultivars (Figure 5 and Figure 7). Our study revealed several candidate genes, among which the expression levels of *BnSBT3.14a* and *BnSBT3.14b* tended to increase in the low-yield material (Figure 5 and Figure S3). Other candidate genes, such as *BnSBT1.4b* and *BnSBT1.4c*, were expressed at higher levels in the high-yield material (Figure 5 and Figure S3). Coincidentally, *SASP (AtSBT1.4)* was reported to be related to the regulation of silique grain number, thereby increasing yield [8,18]. These findings further suggest that the *BnSBT1.4* gene may affect the distribution of photosynthetic products in the silique pericarp, thereby affecting yield in *B. napus*.

The roles of these identified *BnSBTs* are expected to be functionally verified in the future. This comprehensive analysis of *SBT* genes provides a theoretical foundation for studying their functions in plant development and molecular breeding.

## 4. Materials and Methods

### 4.1. Identification of SBT Genes

The protein sequence of the *AtSBT* gene was acquired from The *Arabidopsis* Information Resource (https://www.arabidopsis.org/index.jsp, accessed on 1 August 2023). Identification of the *BnSBT*, *BoSBT*, and *BrSBT* genes was conducted in a three-step procedure. Initially, the *ATSBT* protein sequence served as the query for a BLASTp search [65] against the Brassica database (BRAD, http://brassicadb.org/brad, accessed on 5 August 2023) [66], aiming to identify *SBT* family members. Subsequently, a reverse BLASTp analysis was performed against the *Arabidopsis* protein database for verification. The final step involved using HMMER 3.0 (http://hmmer.org/, accessed on 6 August 2023) to query the Pfam database for the *AtSBT* domain, thereby confirming the presence of the conserved protein domain in the candidate sequences.

### 4.2. Phylogenetic and Evolutionary Analysis

Multiple sequence alignment was performed using MEGA7.0 with default settings. The phylogenetic tree was generated via the neighbor-joining (NJ) method, with a p-distance + G substitution model employed for bootstrap analysis (1000 replicates) [67]. Finally, the tree was visualized using FigureTree (v1.4.4) software.

### 4.3. Chromosomal Locations, Gene Structures, and Conserved Motifs Analysis

Chromosomal locations of *SBT* family members were retrieved from the *Brassica napus* genome database and visualized with Map-Chart2.2 [68]. Gene structures were analyzed and mapped using the Gene Structure Display Server (GSDS) [69], with visualizations generated via TBtools (v1.098). Conserved motifs were examined with the MEME suite [70], with the maximum number of motifs set to 20 (all other parameters remained at default). Protein characteristics, such as molecular weight and isoelectric point, were predicted using the ExPASy ProtParam tool [71] (http://web.expasy.org/protparam/, accessed on 14 August 2023).

Subcellular localization predictions were performed with Plant-mPLoc [72] (http://www.csbio.sjtu.edu.cn/bioinf/plant-multi/, accessed on 16 August 2023), with all parameters set to default.

### 4.4. Promoter Analysis of BnSBTs

*Cis*-regulatory elements were predicted in the 2 kb promoter region upstream of the transcription start site (ATG) for each *BnSBT* gene. These elements were identified using the PlantCARE online database (https://bioinformatics.psb.ugent.be/webtools/plantcare/html/, accessed on 18 August 2023), and their distribution was visualized using TBtools.

### 4.5. RNA-Seq Analysis

To investigate the expression patterns of *BnSBT* genes associated with yield, transcriptome sequencing and analysis were performed on multiple materials with extreme phenotypic traits. The rapeseed materials used included: low thousand-seed weight (L5) and high thousand-seed weight (L6) materials; low seed weight per silique index (L3) and high seed weight per silique index (L4) materials; low initial embryonic number and high initial embryonic number materials; low and high SPS materials; and high harvest index (CQ24, YN24) and low-harvest index (CQ46, YN46) materials from different cultivation locations. The collected tissues included the stem, seed, silique pericarp, lateral branch, main inflorescence, flower bud, pistil, and silique. Samples of rapeseed (*Brassica napus*) stored at −80 °C were sent to Tianjin Novogene Bioinformatics Technology Co., Ltd., for transcriptome sequencing using the Illumina HiSeq 2000 platform. Gene expression was quantified with the FPKM method. The transcriptome data for 140 *BnSBT* genes were extracted, and a heatmap was constructed using TBtools [73]. The data were derived from two different SPSI, TSW, IEN, SPS, and HI (a quantitative yield-related trait) materials across different tissues (seeds, silique pericarp, lateral branch siliques, main inflorescence siliques, and pistils) [32,33].

### 4.6. Identification of Differentially Expressed Genes

Differentially expressed genes (DEGs) were identified following the workflow described in our previous study [74]. Raw RNA-Seq reads were quality-controlled with Trimmomatic (v0.36) [75] to remove adapters and low-quality sequences, aligned to the *Brassica napus* reference genome (v4.1) using STAR (v2.5.3) [76], and counted per gene with feature Counts from Subread (v1.6.0) [77]. Differential expression analysis was performed in R with the edgeR (v3.38.2) package [78], and expression levels were quantified as FPKM using Cuffdiff within Cufflinks [79]. Genes with an FDR-adjusted q-value ≤ 0.05 and |log_2_FC| ≥ 1 were defined as DEGs.

### 4.7. Plant Materials

To investigate the differential expression patterns of *BnSBT* genes in contrasting SPSI materials, seeds of high-SPSI (L4) and low-SPSI (L3) lines were acquired from Chongqing. Plants were field-grown in Chongqing over two consecutive years, with two rows established per experimental plot. Upon maturity, seed yield per plant was measured, and SPSI values were recorded for 10 plants from two biological replicates. Phenotypic data for the H-SPSI and L-SPSI lines are summarized in Table 1 and Figure 5. Data processing, statistical analysis, and graphing were performed using GraphPad 8.5 software. The data are presented as the mean ± SE. Statistical significance of differences between two groups was evaluated using Student’s t-test, with significance levels set as follows: *, *p* < 0.05; **, *p* < 0.01; ***, *p* < 0.001. Tissue samples (seeds and pericarps) were collected at 15 and 35 days after flowering.

### 4.8. RNA Extraction and Validation of RNA-Seq Data by qRT-PCR

Total RNA was isolated from the collected samples with a Total RNA Extractor Kit (Sangon Biotech, Shanghai, China). Subsequently, cDNA was synthesized from the RNA template using a Reverse Transcription Kit (TaKaRa Biotechnology, Dalian, China). Gene-specific primers (Table S7) were designed with Premier 6.0 software [80], and their specificity was verified by local BLAST analysis. The Actin7 gene serves as the endogenous control [81]. Relative gene expression levels were quantified via the 2^−ΔΔCt^ method. Each qRT-PCR assay included three technical replicates. Data visualization was performed with GraphPad Prism 8.5 [82].

## 5. Conclusions

Genome-wide identification and phylogenetic and collinearity analyses were conducted across the whole genomes of the four main Brassicaceae species, revealing that *SBT* genes have undergone gene redundancy and loss during genome evolution, similar to other yield-determining candidate genes reported in our previous publication. Studies on gene structure and conserved motifs suggest that changes in exon–intron structure can alter coding regions, ultimately affecting gene expression and function. The prediction of promoter *cis*-regulatory elements indicates that most *BnSBTs* are associated with hormones and plant growth and development. Moreover, the results of the RNA-Seq analysis and qRT-PCR verification across different yielding cultivars indicated that eight candidate genes showed differential expression, making them ideal candidates for further functional studies.

## Figures and Tables

**Table 1:** Relevant agronomic traits of L-SPSI and H-SPSI materials during the two years of field tests.

Trait	Material	2020	2021	Mean Value	SEM	*p* Value
Seed weight per silique index/% (SPSI)	L-SPSI	51.25	52.59	51.92	0.67	0.00947
	H-SPSI	67.43	70.49	68.96	1.53	
Thousand seed weight/g (TSW)	L-SPSI	3.9	3.96	3.95	0.03	0.00070
	H-SPSI	5.31	5.27	5.29	0.02	
Seed number per silique (SPS)	L-SPSI	20.1	20.4	20.25	0.15	0.00171
	H-SPSI	24.5	24.7	24.6	0.1	

## Data Availability

The information for the ZS11 online database is provided as follows: DOI: 10.3390/ijms21165831; The information for the Arabidopsis Information Resource database is provided as follows: DOI: 10.1093/nar/29.1.102; The RNA-sequencing data were derived from https://www.mdpi.com/1422-0067/24/3/2543 (accessed on 18 August 2023) and https://www.nature.com/articles/srep36452 (accessed on 18 August 2023). The qRT-PCR validation data generated in this study are included in the article and the supplementary material. Further inquiries can be directed to the corresponding author.

## Supplementary Materials

The following supporting information can be downloaded at https://www.mdpi.com/article/10.3390/plants15091318/s1. Figure S1. Comprehensive phylogenetic and *cis*-regulatory element analyses of *BnSBT* genes. Figure S2. Expression patterns of the 140 *BnSBTs* in various tissues in the ZS11 material. Figure S3. Heatmap of the transcriptome data for eight candidate genes in extreme-yield materials. Table S1. Homologous genes among the A and C subgenomes of *B. napus*, *B. rapa*, *B. oleracea,* and their homologs in *Arabidopsis*. Table S2. Numbers of homologous genes among the A and C subgenomes of *Brassica napus*, *Brassica rapa*, *Brassica oleracea*, and their homologs in *Arabidopsis*. Table S3. Analysis of the physicochemical properties of proteins from *SBT* family members of *B. napus*. Table S4. SBT orthologous gene pairs in *Brassica napus*. Table S5. *SBT* orthologous gene pairs in *A. thaliana* compared with those in *B. rapa*, *B. oleracea,* and *Brassica napus*. Table S6. *Cis*-elements in the promoter regions of *BnSBTs*. Table S7. Primers used in this study. Table S8. qRT-PCR analysis of relative expression levels for different *BnSBT* genes (Mean ± SEM).
