# Supplementary material for: Comprehensive Analyses of Serine Protease-like Protease (SBT) in Regulating Yield Characters in Rapeseed (Brassica napus L.)
Source: Plants (Basel). 2026 Apr 25;15(9):1318. doi: 10.3390/plants15091318 (PMC13164763; doi:10.3390/plants15091318)
Supplement: Supplementary file 1 [file plants-15-01318-s001.zip › Supplemental Figures.pdf]

Supplemental Figures

Figure S1. Phylogenetic and *cis*-regulatory element comprehensive analysis of *BnSBT* genes.

Figure S2. The expression patterns of the 140 *BnSBTs* in various tissues in ZS11 material.

Figure S3. Heatmap of transcriptome data for eight candidate genes in extreme yield materials.

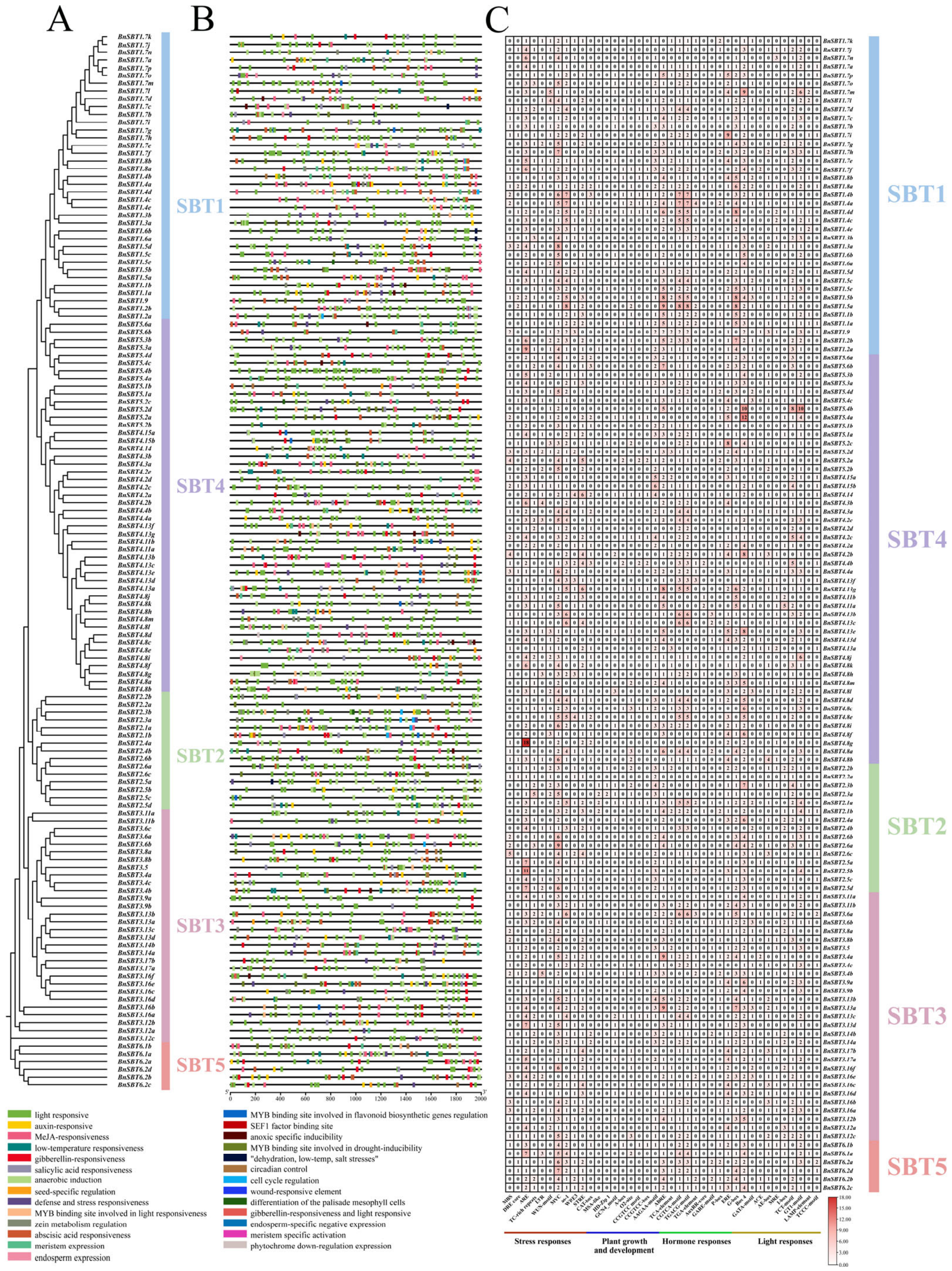

Figure S1: Phylogenetic and cis-regulatory element comprehensive analysis of *BnSBTs*. A: Phylogenetic tree of *BnSBTs*. Different subfamilies are distinguished with colored backgrounds; B: Distribution of cis-regulatory elements in the promoter regions of *BnSBTs*. Different colors and shapes represent specific cis-elements. The legend explains the specific functions of each cis-element; C: Heatmap of the frequency of cis-elements in the promoter regions of *BnSBTs*. The rows represent individual genes, while the columns correspond to different categories of cis-elements. The intensity of red indicates the abundance of a given cis-element in the promoter region of each gene.

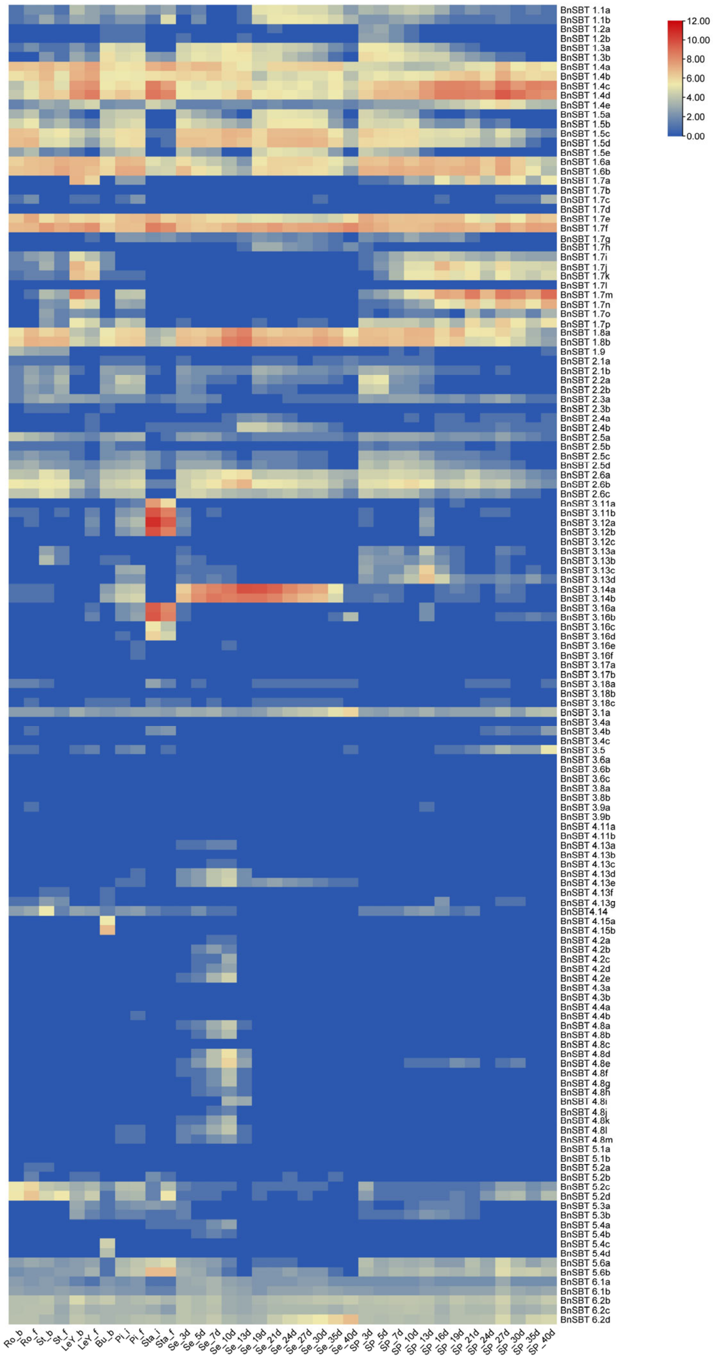

Figure S2. The expression patterns of the 140 *BnSBTs* in various tissues in ZS11 material. Ro_b: Root at budding stage; Ro_f: Root at flowering stage; St_b: Stem at budding stage; St_f: Stem at flowering stage; LeY_b: Leaf at budding stage; LeY_f: Leaf at flowering stage; Bu_b: Bud at budding stage; Pi_i: Pistil at initial flowering stage; Pi_f: Pistil at flowering stage; Sta_i: Stamen at initial flowering stage; Sta_f: Stamen at flowering stage; Se_3d, Se_5d, Se_7d, Se_10d, Se_13d, Se_19d, Se_21d, Se_24d, Se_27d, Se_30d, Se_35d, Se_40d: Seeds at 3, 5, 7, 10, 13, 19, 21, 24, 27, 30, 35, and 40 days after pollination in green pods; SP_3d, SP_5d, SP_7d, SP_10d, SP_13d, SP_16d, SP_19d, SP_21d, SP_24d, SP_27d, SP_30d, SP_35d, SP_40d: Silique pericarps at 3, 5, 7, 10, 13, 16, 19, 21, 24, 27, 30, 35, and 40 days after pollination.

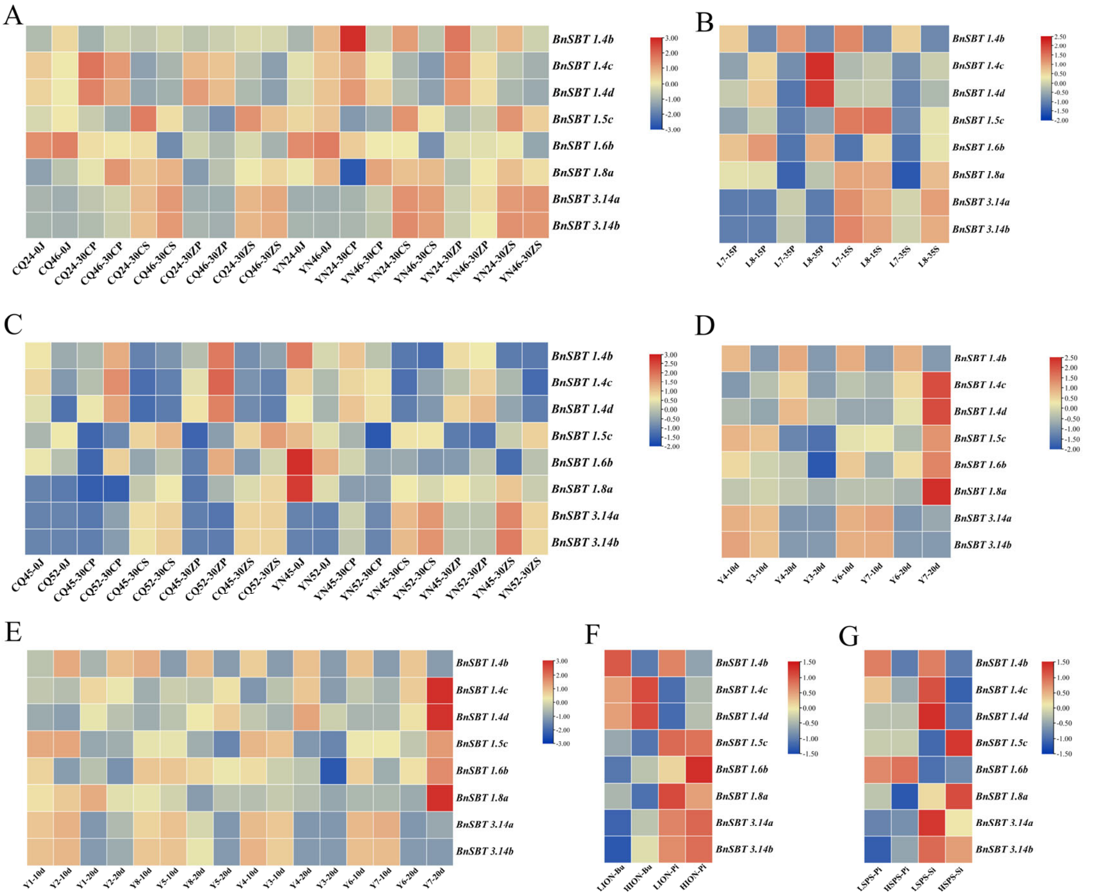

Figure S3. Heatmap of transcriptome data for eight candidate genes in extreme yield materials. A: Harvest index. CQ and YN indicate different cultivation locations. CQ24 and YN24 are high harvest index materials, while CQ46 and YN46 are low harvest index materials. (B) Thousand seed weight (TSW). L7 indicates low TSW, and L8 indicates high TSW. (C) Harvest index. CQ and YN indicate different cultivation locations. CQ45 and YN45 are high harvest index materials, while CQ52 and YN52 are low harvest index materials. (D) and (E) Y2, Y3, Y5, and Y7 are high-yield materials; Y1, Y4, Y6, and Y8 are low-yield materials. (F) Initial embryonic number (IEN). (G) Seed number per silique (SPS). Abbreviations for tissues: J: Stem; S: Seed; P: Silique pericarp; C: Lateral branch; Z: Main inflorescence; L: Flower bud; D: Seed.
